# Analysis of pathogen distribution and risk factors for extended antibiotic course in children with microbiological-based protracted bacterial bronchitis in southwest China

**DOI:** 10.3389/fped.2026.1747356

**Published:** 2026-05-22

**Authors:** Donghai Wang, Su Yang, Yajun Mou, Xingling Pu, Qiumeng Xiang, Jianchuan Chen, Zhou Fu, Jihong Dai

**Affiliations:** 1 Department of Respiratory Disease, Children’s Hospital of Chongqing Medical University, National Clinical Research Center for Children and Adolescents’ Health and Diseases, Ministry of Education Key Laboratory of Child Development and Disorders, Chongqing Key Laboratory of Child Rare Diseases in Infection and Immunity, Chongqing, China; 2 Department of Pediatrics, Longchang City Maternal and Child Health Care Hospital, Longchang, China; 3 Department of Pediatrics, the People’s Hospital of Dejiang City, Tongren, China; 4 Department of Pediatrics, Tengchong City Maternal and Child Health Care Hospital, Tengchong, China; 5 Department of Prophylactic Vaccination, Chongqing Youyoubaobei Woman and Children’s Hospital, Chongqing, China

**Keywords:** corticosteroids, course of antibiotics, influence factors, protracted bacterial bronchitis, streptococcus pneumoniae

## Abstract

**Background:**

Prior studies of protracted bacterial bronchitis (PBB) in children were mainly conducted in high-income areas, and no data from low- and middle-income countries have been published. In this study, we aimed to clarify the outcomes of PBB in a larger cohort in southwest China, focusing on the pathogen distribution and risk factors with an extended course of antibiotics.

**Methods:**

Children with PBB who underwent flexible bronchoscopy were included. Clinical review, cytologic examination, and semiquantitative bacterial culture of bronchoalveolar lavage fluid (BALF) were performed as clinically indicated. Multiple linear regression and decision tree analyses were used to determine factors with an extended course of antibiotics.

**Results:**

The study included 138 children. Two pathogens were detected in 23 BALF samples. *Streptococcus pneumoniae* (Sp) and *Haemophilus influenzae* (Hi) were cultured in 67 and 59 BALF samples, respectively. Sp infection in BALF was an independent factor influencing the duration of the course of antibiotics (*β* = 11.988, *t* = 2.529, *P* = 0.013) according to multiple linear regression. One rule was used to predict an extended course of antibiotics (>4 weeks) with accuracy of 87.54% by decision tree analysis, namely, Sp infection in BALF, percentage of neutrophils in BALF >91%, and use of inhaled corticosteroids (ICS) before admission.

**Conclusion:**

The most prevalent pathogen in children with PBB in southwest China was *S. pneumoniae*, which necessitated a longer course of antibiotics, particularly when paired with a percentage of neutrophils in BALF >91% and administration of ICS prior to admission. Further research of children with PBB in low- and middle-income countries is urgently required.

## 1 Introduction

Protracted bacterial bronchitis (PBB) affects up to 40% of children with chronic wet cough (1), which places a heavy burden on many families (2). Undertreated PBB can lead to relapse and even the life-limiting chronic disease bronchiectasis (3). PBB is mostly diagnosed clinically on the basis of chronic wet cough and a response to 2 weeks of appropriate antibiotic treatment (also termed PBB-clinical) (1). The definitive diagnosis can be established by positive bacterial sputum or bronchoalveolar lavage fluid (BALF) cultures, also called microbiological-based PBB or PBB-micro (4). PBB-extended is defined as either PBB-clinical or PBB-micro, with cough resolution occurring only after a 4-week course of antibiotics (4). A recent multicenter, double-blind, randomized controlled trial compared cough resolution between children with PBB who were treated with oral amoxicillin-clavulanate for 2 or 4 weeks and found no significant difference between the two groups (5). Nevertheless, some children require longer antibiotic treatment in clinical practice (6–8). Antibiotic courses of 2, 2–4, and 4–6 weeks are recommended by the American CHEST Cough Guidelines (9), the European Respiratory Society (1), and the British Thoracic Society (10), respectively. Therefore, more evidence is needed to establish the optimal duration of antibiotic treatment, and it is essential to determine which children with PBB might need an extended course of antibiotics.

As bronchoscopy is an invasive procedure, it is not feasible in routine clinical practice and should not be regarded as a standard component of the PBB diagnostic workup. However, it may be considered in selected cases. Haemophilus influenzae (Hi) [usually non-typeable Hi (NTHi) strains] is consistently the most common bacterial pathogen isolated from BALF in western countries, followed by Streptococcus pneumoniae (Sp) and Moraxella catarrhalis (Mc) (1). In western countries, most children receive pneumococcal vaccines (11). These vaccines influence the epidemiology of PBB by conferring individual immunity and concurrently altering serotype circulation in immunized populations (12). In PBB, recurrent exacerbations and lower airway Hi infection are associated with future bronchiectasis (3, 13). Therefore, lower airway Hi infection must be appropriately treated with antibiotics. In low- and middle-income countries, adoption of pneumococcal vaccines into national immunization programs is limited (14). In China, pneumococcal vaccination coverage is lower than in high-income countries, and vaccines are available for private purchase, particularly those living in western China (15). It is unclear whether Hi is the most prevalent bacterial pathogen in children with PBB in western China and which duration of antibiotic treatment is optimal.

More data on PBB in China are required, and only a few studies have conducted follow-up of 4 weeks or longer (16, 17). A survey of Chinese pediatricians showed that antibiotic prescribing practices and diagnostic approaches for recurrent PBB remain suboptimal (18). The optimal duration of antibiotic treatment, its influencing factors, and the long-term prognosis of children with PBB in China are unknown. We performed a retrospective study using clinical records to describe the clinical features, bacterial distribution and to determine factors associated with the extended antibiotic treatment in children with PBB who were referred to the Children's Regional Medical Center in southwest China. In addition, children with PBB were followed up to understand the prognosis of this disease in southwest China.

## 2 Materials and methods

### 2.1 Study setting and ethical approval

The Children's Hospital of Chongqing Medical University is a regional medical center for children in southwest China. It provides specialist medical services mainly in Chongqing, Sichuan, Guizhou, and Yunnan provinces, which encompass more than 2 million km^2^. This study was approved by the Ethics Committee of the Children's Hospital of Chongqing Medical University (approval No.: 2023-Y-419) and all collected data were kept strictly confidential.

### 2.2 Study subjects and process

We retrospectively investigated hospitalized children with chronic wet cough who were admitted to the Children's Hospital of Chongqing Medical University from January 2018 to December 2024. Our inclusion criteria were (1) chronic wet cough lasting >4 weeks; (2) chest x-rays showed no abnormality or chest high-resolution computed tomography (HRCT) demonstrated one of the following: no abnormality, mild bronchial wall thickening, an inhomogeneous density of air trapping or localized minor peribronchial infiltrates. Children with bronchiectasis, atelectasis, pulmonary mass, or other significant structural abnormalities on chest HRCT were also excluded; (3) there was evidence of lower respiratory infection (positive semiquantitative bacterial culture of BALF); (4) resolution of cough after completion of an appropriate antibiotic course, as documented in discharge records and first outpatient follow-up after discharge. Patients with tracheomalacia, bronchomalacia, tracheostenosis, bronchiarctia, and other underlying disease (e.g., leukemia, epilepsy, primary immunodeficiency, and primary ciliary dyskinesia) were excluded. During hospitalization, amoxicillin clavulanate or appropriate antibiotics (according to the bacterial culture and antibiotic sensitivity testing results) were administered intravenously. Once the cough was substantially improved, patients were transitioned to oral antibiotics. Outpatient follow-up records were reviewed. If outpatient follow-up records were unavailable, parents were contacted by telephone. Data were extracted from the hospital's electronic medical record system. Children with PBB were followed up by telephone calls or outpatient department visits for 1–4 years to assess relapse and bronchiectasis.

### 2.3 Data collection and research variables

Demographic data and clinical characteristics were collected retrospectively. The primary outcome variable was the total duration of antibiotic treatment in days, encompassing both pre- and post-admission courses and including both intravenous and oral antibiotics. All relevant clinical data, including demographic characteristics (sex and age), clinical presentation (duration of cough and wheeze), complications (nasosinusitis), and use of inhaled corticosteroids (ICS) before admission were recorded. The results of procalcitonin (PCT) examination, complete blood count, cytologic examination of BALF, and semiquantitative bacterial culture of BALF were also documented. The pneumococcal vaccination records including the 13-valent pneumococcal conjugate vaccine (PCV13, four doses) and 23-valent pneumococcal polysaccharide vaccine (PPSV23, one dose) were also documented by the vaccination status database of each province. Relapse was defined as recurrence of chronic wet cough lasting more than 4 weeks after documented resolution upon completion of antibiotic treatment and was identified through review of outpatient clinic records or telephone follow-up with parents. Bronchiectasis was identified by chest HRCT performed during follow-up in children with recurrent or persistent symptoms.

### 2.4 Data analysis

Categorical data (sex, wheeze, PCV13, PPSV23, ICS, nasosinusitis, and bacterial pathogens) were described by the absolute number, frequency, and proportion. Continuous variables (age, duration of cough, PCT, various indexes in blood and BALF routine examinations, and days of antibiotic treatment) were described by the median and interquartile range (IQR) in descriptive analysis. The Shapiro–Wilk test was used to verify the normality of continuous variables, which were all non-normally distributed. Due to the limited number of events, relapse of chronic wet cough and bronchiectasis were analyzed descriptively only. Univariate analyses were performed to examine associations between candidate predictor variables and the outcome variable. For categorical variables, the Mann–Whitney *U* test was used to compare the groups in terms of days of antibiotic treatment. For continuous variables, Spearman's correlation was used to compare the patients in terms of days of antibiotic treatment. Multiple linear regression analysis was performed to determine which factors influenced the days of antibiotic treatment (*P* < 0.1 in univariate analysis). The *P* < 0.1 threshold, rather than the conventional *P* < 0.05, was adopted to reduce the risk of prematurely excluding predictors of potential clinical relevance, a criterion widely used in multivariable modeling particularly in studies with relatively modest sample sizes. Patients were divided into two groups according to the days of antibiotic treatment, with a boundary of 4 weeks. In univariate analysis, the candidate variables with *P* < 0.1 were selected for inclusion in the decision tree model. Decision tree analysis (algorithm = classification and regression tree, CART) was used to identify factors closely linked to antibiotic treatment for longer than 4 weeks. 70% of patients were randomly assigned to the training set and the remaining 30% comprised the test set for internal validation. Then, overall accuracy was determined to verify the predictive value of the decision tree model. Statistical analyses were performed using SPSS 26.0 and SPSS Modeler 18.0 (IBM Corp, Armonk, NY, USA) with a significance level of *P* < 0.05.

## 3 Results

### 3.1 Patient demographic and clinical characteristics

**Figure 1:**
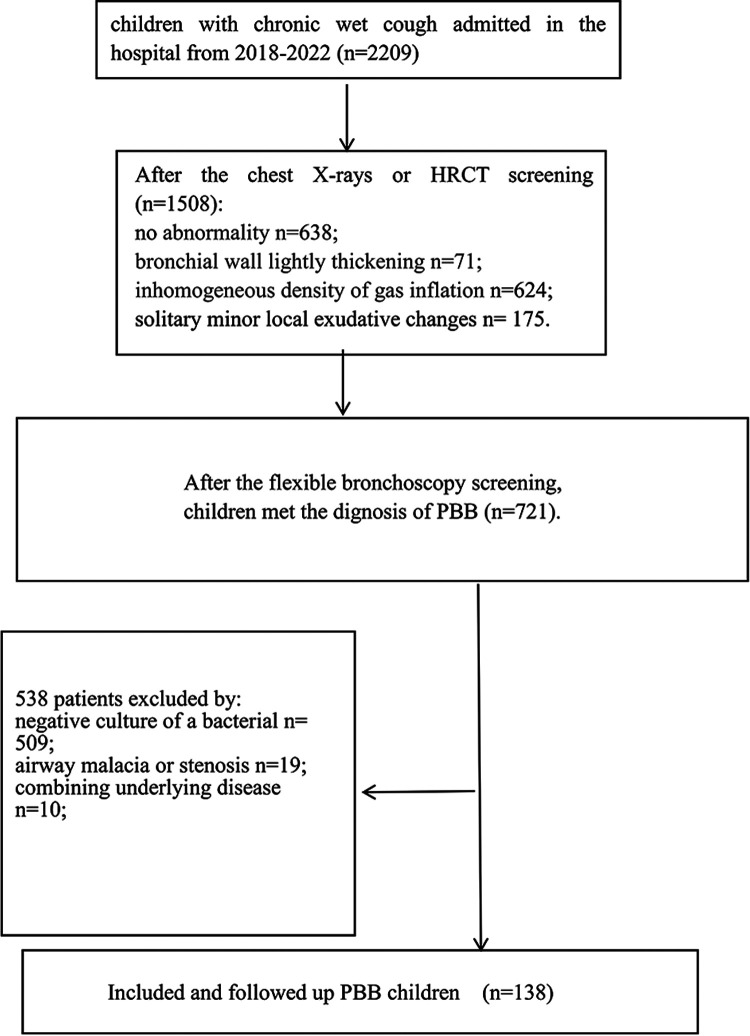
The flow chart of enrollment of the study population.

A total of 2,209 children with chronic wet cough were enrolled in this study, and 138 children diagnosed with PBB were included and followed up. Figure 1 shows the study enrollment flowchart. The sample included 79 boys (57.25%) and 59 girls (42.75%) with a median age of 2.25 (IQR: 1.75–4.00) years. The number of days of antibiotic treatment ranged from 14 to 94 with a median of 24 days (IQR: 14.5–35 days), and 47 (34.06%) children were treated with antibiotics for longer than 4 weeks. Overall, 7 (5.07%) children received four doses of PCV13 and 131 (94.93%) children were unvaccinated; 57 (41.30%) children were immunized with PPSV23 and 81 (58.70%) children were unvaccinated. In total, 63 (45.65%) children had wheezing and 75 (54.35%) children had sinusitis. Cytology of BALF samples was dominated by neutrophils. All children initially received intravenous amoxicillin-clavulanate during hospitalization, followed by oral antibiotics after discharge. No child received concurrent dual antibiotic therapy. Among the 47 children with an extended antibiotic course, 7 (5.1%) were switched to oral linezolid and 5 (3.6%) to oral levofloxacin after 4 weeks of persistent symptoms, based on parental consent and antibiotic sensitivity results. The median duration of follow-up was 1.78 years, and 11 (7.97%) children were lost to follow-up. Regarding patient outcomes, of the 127 children who completed follow-up, nine (6.50%) children experienced 1–3 relapses of chronic wet cough and 2 (1.45%) children developed bronchiectasis, confirmed by chest HRCT. Table 1 shows the basic characteristics of the patients and univariate analysis of the relationship of each factor with the outcome variable.

**Table 1:** Basic characteristics of the study participants and univariate analysis of the relationship of each factor with days of antibiotic treatment.

Variable	Cases (%) or median (*P*_25_, *P*_75_)	Days of antibiotic treatment		
		Median (*P*_25_, *P*_75_)	*P* value	Z or r value
PPSV23			0.218	−1.232
Unvaccinated	81 (58.70%)	28 (16, 36)		
Immunized	57 (41.30%)	21 (14, 33)		
PCV13			**0** **.** **06**	−1.882
Unvaccinated	131 (94.93%)	25 (16, 35)		
Vaccinated	7 (5.07%)	14 (14, 14)		
ICS			**0** **.** **091**	−1.692
No	85 (61.59%)	21 (14, 32.5)		
Yes	53 (38.41%)	28 (17, 39.5)		
Sex			0.495	−0.683
Girl	59 (42.75%)	28 (16, 35)		
Boy	79 (57.25%)	21 (14, 33)		
Nasosinusitis			**0** **.** **042**	−2.034
No	63 (45.65%)	21 (14, 31)		
Yes	75 (54.35%)	28 (17, 35)		
Wheeze			0.722	−0.356
No	75 (54.35%)	21 (16, 35)		
Yes	63 (45.65%)	28 (14, 33)		
*Haemophilus influenzae*			**0** **.** **064**	−1.854
No	79 (57.25%)	21 (14, 33)		
Yes	59 (42.75%)	28 (19, 37)		
*Moraxella catarrhalis*			**0** **.** **001**	−3.244
No	109 (78.99%)	28 (17, 35)		
Yes	29 (21.01%)	16 (14, 24.5)		
*Staphylococcus aureus*			0.15	−1.44
No	132 (95.65%)	24 (16, 35)		
Yes	6 (4.35%)	16.5 (14, 28.75)		
*Streptococcus pneumoniae*			**<0** **.** **001**	−4.321
No	71 (51.45%)	20 (14, 28)		
Yes	67 (48.55%)	30 (21, 45)		
*Haemophilus influenzae* and *Streptococcus pneumoniae*			**<0** **.** **001**	−4.333
No	123 (89.13%)	21 (14, 31)		
Yes	15 (10.87%)	45 (34, 59)		
Age	2.25 (3.75, 5)	24 (14.5, 35)	**0** **.** **053**	0.165
Duration of cough (days)	3 (1.875, 6)	24 (14.5, 35)	0.990	0.001
Peripheral white blood cell count (10^9^/L)	8.56 (7.33, 10.47)	24 (14.5, 35)	0.174	−0.116
Neutrophil % in peripheral blood	46 (35, 54)	24 (14.5, 35)	0.456	0.064
Procalcitonin (ng/mL)	0.046 (0.03, 0.07)	24 (14.5, 35)	0.360	−0.078
Karyocytes in BALF (10^6^/L)	3,847 (1,185, 7,500.25)	24 (14.5, 35)	0.213	0.107
Neutrophil % in BALF	68 (48, 84.25)	24 (14.5, 35)	**0** **.** **003**	0.250
Macrophage % in BALF	12 (4, 23.5)	24 (14.5, 35)	0.582	−0.047
Lymphocyte % in BALF	9 (4, 13)	24 (14.5, 35)	0.128	−0.13
Eosinophil % in BALF	1 (0, 2)	24 (14.5, 35)	0.235	0.102

ICS, Inhaled corticosteroids; PCV13, 13-valent pneumococcal conjugate vaccine; PPSV23, 23-valent pneumococcal polysaccharide vaccine; BALF, Bronchoalveolar lavage fluid; PCT, procalcitonin; IQR, Interquartile range.

Bold values in the *P* column indicate *P* < 0.1.

### 3.2 Pathogen distribution in BALF

**Figure 2:**
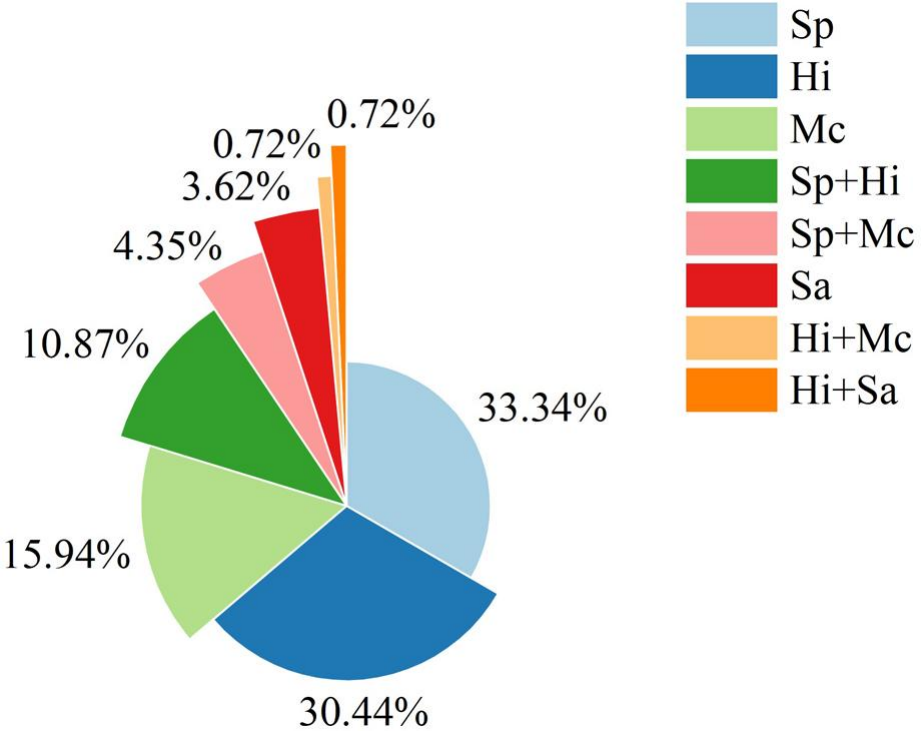
The distribution of pathogens identified in BALF cultures among 138 children with PBB. Sp, *Streptococcus pneumoniae*; Hi, *Haemophilus influenzae*; Mc, *Moraxella catarrhalis*; Sa, *Staphylococcus aureus*; Sp + Hi, co-infection with *S. pneumoniae* and *H. influenzae*; Sp + Mc, co-infection with *S. pneumoniae* and *M. catarrhalis*; Hi + Mc, co-infection with *H. influenzae* and *M. catarrhalis*; Hi + Sa, co-infection with *H. influenzae* and *S. aureus*.

Among 138 BALF samples, 115 yielded a single pathogen and 23 yielded two pathogens, resulting in 161 pathogen isolates in total. Of these, 46 (33.33%) children cultured Sp, 42 (30.43%) children cultured Hi, 22 (15.94%) children cultured Mc, 5 (3.62%) children cultured Staphylococcus aureus (Sa), 15 (10.87%) children cultured Sp and Hi, 6 (4.35%) children cultured Sp and Mc, 1 (0.72%) child cultured Hi and Mc, and 1 (0.72%) child cultured Hi and Sa. Hi and Sp were cultured in 59 (42.75%) and 67 (48.55%) of all samples, respectively (both were cultured in 10.87% of samples). The distribution of pathogens identified in BALF cultures is illustrated in Figure 2.

### 3.3 Multiple linear regression of days of antibiotic treatment

A multiple linear regression model was constructed using age, nasosinusitis, PCV13, percentages of neutrophils and columnar cells in BALF, Hi, Mc, Sp, Hi and Sp, and ICS as independent variables and days of antibiotic treatment as the dependent variable (Table 2). There was a positive correlation between Sp infection in BALF and days of antibiotic treatment (*β* = 11.988, *t* = 2.529, *P* = 0.013). Sp infection was an independent factor affecting days of antibiotic treatment.

**Table 2:** Coefficients of the multiple linear regression analysis model with days of antibiotic treatment as the dependent variable.

Variable	Unstandardized coefficient		Standardized coefficient	*P*	VIF
	*β*	Standard error	*β*		
Nasosinusitis	3.890	2.502	0.117	0.122	1.052
Neutrophil % in BALF	0.117	0.064	0.178	0.071	1.784
*Moraxella catarrhalis*	0.106	4.510	0.003	0.981	2.287
*Streptococcus pneumoniae*	11.988	4.741	0.360	**0** **.** **013**	3.805
*Haemophilus influenzae*	6.078	5.061	0.181	0.232	4.248
*Haemophilus influenzae* and *Streptococcus pneumoniae*	7.518	6.340	0.141	0.238	2.639
PCV13	−1.505	5.960	−0.020	0.801	1.159
ICS	2.162	2.720	0.063	0.428	1.186
Age	0.810	0.621	0.106	0.194	1.240

PCV13, 13-valent pneumococcal conjugate vaccine; ICS, inhaled corticosteroids; BALF, bronchoalveolar lavage fluid; VIF, variance inflation factor.

Bold values in the *P* column indicate *P* < 0.05.

### 3.4 Decision tree analysis

**Figure 3:**
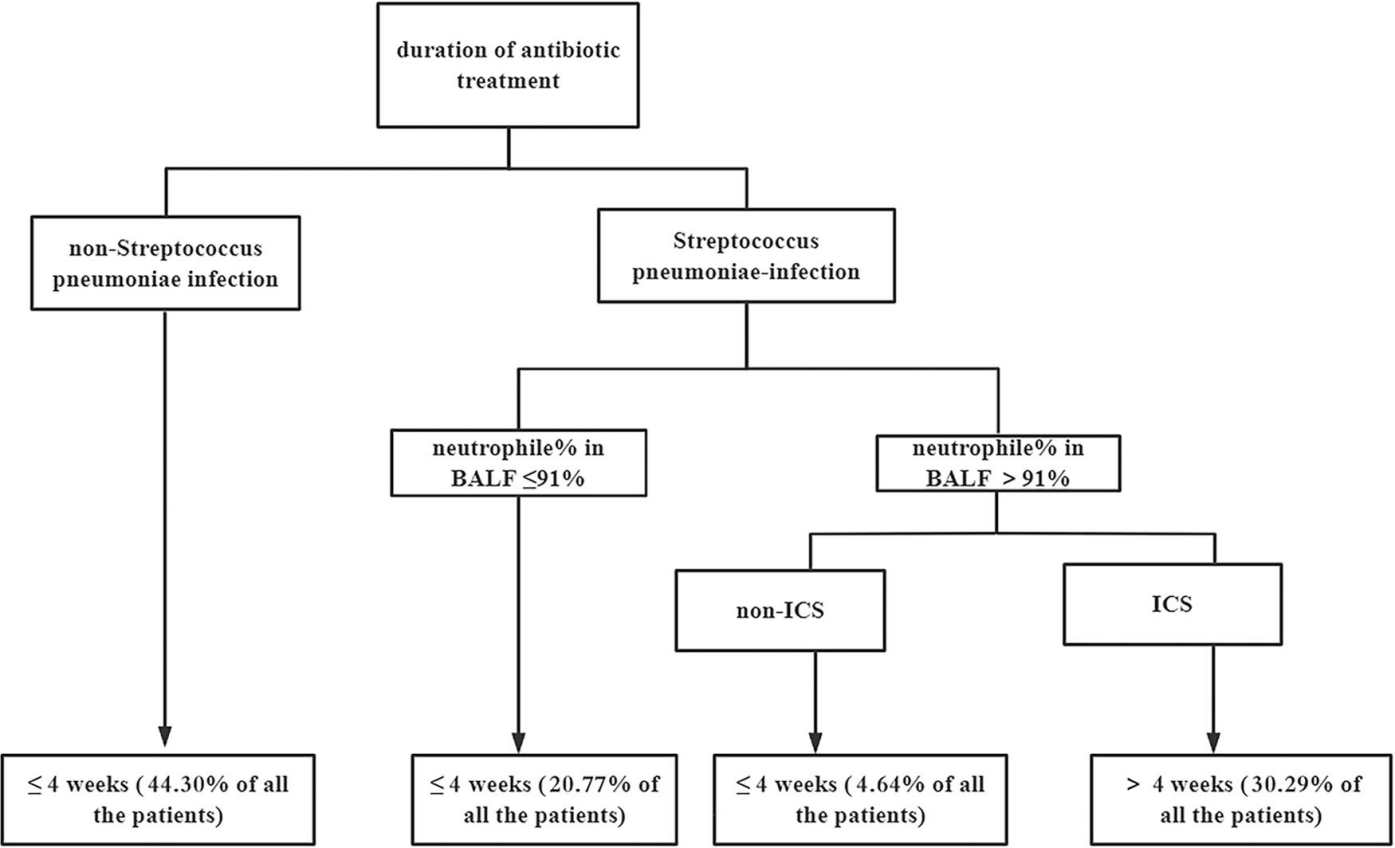
The decision tree. ICS, inhaled corticosteroids; BALF, bronchoalveolar lavage fluid.

Patients with PBB were analyzed via decision tree analysis. According to days of antibiotic treatment, 47 (34.06%) and 91 (65.94%) children were assigned to the PBB-extended (>4 weeks) and PBB (≤4 weeks) groups, respectively. The baseline characteristics of the two groups are presented in Table 3. Compared with the PBB group, children in the PBB-extended group had a significantly higher proportion of Sp infection (83.0% vs. 30.8%, *P* < 0.001), a higher rate of nasosinusitis (68.1% vs. 47.3%, *P* = 0.042), a higher percentage of BALF neutrophils (median 79% vs. 63%, *P* = 0.003), and a higher rate of Sp + Hi co-infection (27.7% vs. 2.2%, *P* < 0.001). In contrast, Mc infection was more frequent in the PBB group (27.5% vs. 8.5%, *P* = 0.001). Age, sex, duration of cough, wheeze, ICS use, vaccination status, and peripheral blood parameters did not differ significantly between the two groups. In total, 70% of patients were randomly assigned to the training set and the remaining 30% comprised the test set. A decision tree model was constructed using variables with *P* < 0.1 in univariate analysis. The three decision variables identified were Sp infection, BALF neutrophil percentage, and ICS use before admission. The decision tree is presented in Figure 3. The root node of the decision tree model was Sp infection. Only one rule predicted an extended antibiotic course (>4 weeks), namely, Sp infection in BALF, percentage of neutrophils in BALF >91%, and use of ICS before admission. The other three rules predicted a short-term antibiotic course (≤4 weeks). According to these rules, the test set data were predicted and accuracy was 87.54%. The predictive value of the decision rules was confirmed in the test set.

**Table 3:** Baseline characteristics of children in the PBB group (≤4 weeks) and PBB-extended group (>4 weeks).

Characteristic	PBB group (≤4 weeks, *n* = 91)	PBB-extended (>4 weeks, *n* = 47)	*P* value
Age, years, median (IQR)	2.08 (1.67–3.83)	3.00 (1.92–4.50)	0.053
Male sex, *n* (%)	52 (57.1)	27 (57.4)	0.495
Duration of cough before admission, months, median (IQR)	3.0 (2.0–5.0)	3.0 (1.5–7.0)	0.990
Wheeze, *n* (%)	41 (45.1)	22 (46.8)	0.722
Nasosinusitis, *n* (%)	43 (47.3)	32 (68.1)	0.042
ICS use before admission, *n* (%)	31 (34.1)	22 (46.8)	0.091
PCV13 vaccinated, *n* (%)	6 (6.6)	1 (2.1)	0.060
PPSV23 vaccinated, *n* (%)	39 (42.9)	18 (38.3)	0.218
Peripheral white blood cell count (10⁹/L), median (IQR)	8.32 (7.10–10.01)	8.94 (7.68–11.03)	0.174
Neutrophil % in peripheral blood, median (IQR)	44 (34–52)	49 (37–56)	0.456
Procalcitonin (ng/mL), median (IQR)	0.044 (0.03–0.065)	0.051 (0.035–0.078)	0.360
BALF neutrophils, %, median (IQR)	63 (43–79)	79 (67–91)	0.003
*S. pneumoniae* positive, *n* (%)	28 (30.8)	39 (83.0)	<0.001
*H. influenzae* positive, *n* (%)	32 (35.2)	27 (57.4)	0.064
*M. catarrhalis* positive, *n* (%)	25 (27.5)	4 (8.5)	0.001
*S. aureus* positive, *n* (%)	5 (5.5)	1 (2.1)	0.150
Co-infection Sp + Hi, *n* (%)	2 (2.2)	13 (27.7)	<0.001

ICS, inhaled corticosteroids; PCV13, 13-valent pneumococcal conjugate vaccine; PPSV23, 23-valent pneumococcal polysaccharide vaccine; BALF, bronchoalveolar lavage fluid.

## 4 Discussion

The data presented in this study show that Sp was the most common pathogen in children with PBB in southwest China and required a longer course of antibiotics. To the best of our knowledge, this is the first time that cohort data have been used to identify factors associated with the course of antibiotics in children with PBB in a low- or middle-income country with a lower level of pneumococcal vaccination coverage. This was also a long-term follow-up study of children with PBB focusing on relapse of chronic wet cough and bronchiectasis. Our findings offer clinically relevant guidance for the management and prognostic assessment of children with PBB in developing countries.

Only children confirmed to have PBB by flexible bronchoscopy and analysis of BALF were included to minimize ambiguity about the diagnosis. Sp and Hi were cultured in 67 (48.55%) and 59 (42.75%) BALF samples, respectively, and other organisms detected were Mc and occasionally Sa. Sp was the most common pathogen, consistent with two Chinese retrospective studies (19, 20), but this contrasts with the dominance of NTHi reported in high-income regions (7, 21, 22). The most likely explanation for this discrepancy is the substantially lower pneumococcal conjugate vaccine coverage in China compared with developed countries. The rate of immunization with a pneumococcal vaccine was higher than 90% in studies in Australia (13, 22), but only seven children were vaccinated with PCV13 in this study. Evidence suggests that the decrease of Sp-mediated and increase of NTHi-mediated acute otitis media (AOM) are associated with introduction of pneumococcal vaccines (23, 24). The pathogen distribution is similar in AOM and PBB (25); therefore, we speculate that the dominance of NTHi in children with PBB in developed countries is associated with the introduction of pneumococcal vaccines. Additionally, clinical studies show that pneumococcal non-vaccine serotypes have replaced those targeted by pneumococcal vaccines as the etiological cause of PBB in current cases (12, 22). In this study, the incidences of Hi and Sp infections were compared between children who were inoculated with PPSV23 and those who were not using the chi-square test (statistical analysis could not be performed for PCV13 due to limited data), and no statistical difference was found (*P* = 0.27 and *P* = 0.19). This result is inconsistent with our previous hypothesis, which might be due to the difference in individual immunity and a concurrent change of serotype circulation in immunized populations. Biofilms exist and therefore bacteria are often not recovered by culture (26), and the positivity rate of BALF culture is low. The small number of included samples do not adequately represent immunity of the population. Therefore, this difference in vaccine coverage may partially explain the distinct pathogen distribution observed in our cohort, but further robust epidemiological studies are required to confirm this hypothesis.

Previous studies evaluated cough resolution and recurrence of chronic wet cough with a fixed course of antibiotics (5, 6, 8). However, children with PBB often require prolonged antibiotic treatment. Meanwhile, longer courses of antibiotics are associated with increased cost, potential adverse effects, an increased risk of antibiotic resistance, and poorer adherence. To improve parental adherence, the anticipated duration of antibiotic treatment should be estimated as accurately as possible at the outset. This study aimed to explore factors that influence the course of antibiotics. Univariate analysis and multivariate linear regression analyses revealed that Sp infection was linked with an extended course of antibiotics. According to drug sensitivity tests, Sp in all BALF samples was sensitive to amoxicillin-clavulanate. We hypothesize that biofilm formation decreases antibiotic efficacy, thereby prolonging the required course of treatment (27). Through persister cells that survive the first exposure, biofilms play a significant role in survival of bacteria after drug treatment and can facilitate antibiotic resistance (28). *In vivo* and *in vitro* studies have demonstrated that Sp is capable of forming biofilms. Even at concentrations higher than their minimum inhibitory concentrations, some commonly used antibiotics, including amoxicillin, erythromycin, and levofloxacin, have weaker antimicrobial effects on strains that produce biofilms than on strains that are planktonic, according to an *in vitro* resistance study of Sp (29). In this study, the majority of children who cultured Sp experienced complete resolution of wet cough with a prolonged course of antibiotics. A possible explanation for this result is that longer courses of antibiotics might be more likely to overcome biofilm resistance, resulting in higher rates of eradication. Another possible explanation is that longer antibiotic courses allow the inflamed and denuded ciliated epithelium to recover, which helps to clear respiratory secretions, resulting in complete resolution of wet cough. This was not a randomized controlled study and the sample size was limited; therefore, we could not pinpoint the exact explanation for this result. Hi and Mc can also produce biofilms; they live together in biofilms in various respiratory tract diseases, such as pneumonia, otitis media, and bronchitis (30). We hypothesize that Sp is more capable of generating biofilms than Hi and Mc. However, the capacities of these three bacteria to generate biofilms were not compared. To confirm this theory, additional experiments are needed. We also found a high rate of co-infection for Sp, particularly with Hi (15/138). On the mucosal surface of the middle ear in mice, Hi increases the formation and persistence of pneumococcal biofilms (31). Perhaps co-infection with Hi was another factor in why PBB children with Sp culture needed an extended antibiotic course. Notably, several variables that showed statistically significant associations with antibiotic duration in univariate analysis—namely, Mc infection (*P* = 0.001), nasosinusitis (*P* = 0.042), and co-infection with Hi and Sp (*P* < 0.001)—did not retain independent significance in the multivariate model and warrant further discussion. Mc infection was paradoxically associated with a shorter antibiotic course in univariate analysis (median 16 days vs. 28 days in non-Mc cases), and its effect became negligible after adjustment for Sp infection (*β* = 0.106, *P* = 0.981). This likely reflects the tendency for Mc to be detected in children without concurrent Sp infection; once Sp status is accounted for, Mc does not independently influence treatment duration. Whether Mc confers a less virulent or more antibiotic-susceptible lower airway infection in children with PBB deserves dedicated investigation. Similarly, nasosinusitis showed a significant univariate association with longer antibiotic courses (*P* = 0.042) but lost significance in the multivariate model (*P* = 0.122), suggesting that this association may be partly confounded by co-occurring Sp infection, as Sp is a well-recognized etiological agent of both sinusitis and lower respiratory tract infection. Co-infection with Hi and Sp was the strongest univariate predictor of antibiotic duration (median 45 vs. 21 days without co-infection, *P* < 0.001), yet this association was attenuated in the multivariate model (*P* = 0.238), likely because of multicollinearity between the co-infection variable and the Sp-alone variable (VIF = 2.639). The markedly prolonged antibiotic courses observed in Hi + Sp co-infected children nonetheless suggest a clinically important synergistic effect, consistent with experimental evidence that Hi enhances pneumococcal biofilm formation and persistence on mucosal surfaces (31). The statistical attenuation in the multivariate model should therefore not be interpreted as evidence that co-infection is clinically unimportant; rather, the limited number of co-infected children (*n* = 15) constrained statistical power, and larger prospective studies are needed to clarify the independent contribution of Hi + Sp co-infection to antibiotic course duration.

Univariate and multiple linear regression analyses have been used to explore factors that influence the course of antibiotics in children with PBB; however, they have not directly provided suggestions for good decision-making. Decision tree analysis is a data mining method for classification data that is used to develop algorithms for predicting results and to generate corresponding rules for classifying samples (32). In our study, four rules for evaluating the duration of the course of antibiotics (with a boundary of 4 weeks) were established. We then predicted the duration of the course of antibiotics in the test set using these four rules and achieved good accuracy (87.54%). Decision tree analysis can objectively predict the course of antibiotics in each patient and avoid subjectivity. In addition, it can boost the confidence of parents and reduce the economic expenditures associated with repeated referrals. However, as this accuracy estimate was derived from an internal 70/30 split validation, it may be optimistic, and the model requires external validation in an independent cohort before its clinical utility can be confirmed.

Besides infection of Sp, the percentage of neutrophils in BALF and use of ICS before admission were the other decision variables that had the strongest discriminative ability in decision tree analysis. A higher percentage of neutrophils in BALF predicted an extended course of antibiotics, which suggests that the course of antibiotics is related to the degree of airway inflammation. Leukocyte elastase secreted by neutrophils is a key mediator of airway injury, contributing to epithelial damage, goblet cell hyperplasia, and excessive mucus production (33). Neutrophils are significantly elevated among BALF cells of children with PBB (34), and airway neutrophil infiltration is involved in the pathogenesis of PBB (35). Stockley et al. (36) found that the color depth of airway secretions is correlated with inflammatory activity, whereby the darker the color, the more severe the airway inflammatory response and the greater the neutrophils burden in the airways. This indicates that the more severe the airway inflammation, the longer the required course of antibiotics. In this study, 53 (38.41%) children had been treated with ICS prior to admission, regardless of whether a confirmed diagnosis of asthma had been established. These data are consistent with those reported from a referral hospital in Australia (38.3%) (37). Bronchoscopy revealed that 48.5% of 33 children with persistent wheeze and no response to ICS had a bacterial airway infection; meanwhile, cytology of BALF showed that neutrophil airway inflammation is the leading cause of no response to inhaled glucocorticoids (38). Decision tree analysis identified ICS use before admission as a factor associated with a prolonged antibiotic course. On the one hand, use of ICS may have delayed antibiotic therapy and potentially contributed to biofilm formation. On the other hand, Hi can respond directly to corticosteroid treatment in the airway, potentially influencing biofilm formation, persistence, and the efficacy of antibiotic treatment (39). Additionally, regular use of ICS is associated with an increased risk of oropharyngeal colonization by Sp in children (40). However, it should also be noted that ICS use in this cohort may reflect underlying disease severity or a misdiagnosis of asthma rather than a direct causal effect on antibiotic duration, and this possibility cannot be excluded in the current retrospective study. Therefore, the observed association between ICS use and prolonged antibiotic course should be interpreted with caution, and whether this reflects a direct pharmacological effect on pathogenic bacteria and biofilms or is mediated by confounding disease characteristics warrants further prospective investigation.

In this study, 2 children developed bronchiectasis during 1–4 years of follow-up and only nine children suffered relapses. These rates are significantly lower than those in previous studies (6, 13). This may be due to several reasons. First, we only included children clinically diagnosed with PBB who had positive BALF cultures and excluded children with underlying diseases and airway abnormalities. If children are misdiagnosed or have an underlying disease (cystic fibrosis, primary immunodeficiency, or primary ciliary dyskinesia), they are prone to develop bronchiectasis due to repeated infections. Second, we used courses of antibiotics sufficient to completely resolve wet cough. Evidence suggests that the initial antibiotic course duration is associated with recurrent PBB relapse (8). Finally, few data have been reported from Asia and Africa, and it remains unclear whether racial or ethnic differences influence PBB outcomes, as has been observed in cystic fibrosis. However, these low rates should also be interpreted with caution, as they may partly reflect underreporting or the inherent limitations of telephone-based follow-up, through which mild or transient relapses may not have been consistently captured. Parents contacted by telephone may not have recognized or reported symptoms that did not lead to a hospital visit, potentially leading to underestimation of true relapse rates.

Our study has several limitations that must be considered. More than 700 children were clinically diagnosed with PBB in our hospital during the study period, but we only included those with positive BALF cultures. Restricting inclusion to bronchoscopy-confirmed PBB-micro cases introduces a selection bias toward more severe presentations and excludes PBB-clinical. The findings, particularly the pathogen distribution and antibiotic course data, may therefore not be generalizable to the broader PBB population, especially in primary care settings where bronchoscopy is not routinely available. More children could be included if metagenomic next-generation sequencing was used instead of BALF culture. Use of more samples might help to conclude that Sp is the most common pathogen due to low pneumococcal vaccine coverage. Furthermore, we did not identify the serotypes of Sp and Hi to assess the clinical effect of vaccination and did not test biofilms. Additionally, we ignored the impact of viruses on children with PBB and we did not test BALF for common viruses. Different types of viruses have been detected in BALF of children with PBB, although the clinical significance is unclear (41). Finally, this was a single-center retrospective study and the results may not reflect the general population. Furthermore, the use of a liberal *P* < 0.1 threshold for variable selection into the multivariate model may have introduced false-positive associations, particularly for variables with borderline significance such as PCV13 vaccination status and ICS use.

## 5 Conclusion

Sp is the most common pathogen in the lower respiratory tract of children with PBB in China, in contrast with Hi in high-income regions. Sp infections often predict an extended course of antibiotics for children with PBB, especially when combined with a percentage of neutrophils in BALF >91% and use of ICS before admission. Children with PBB in China generally have a good prognosis, but some are at risk of recurrence and bronchiectasis. Based on our findings, we postulate that coverage of pneumococcal vaccines may affect the bacterial distribution and duration of antibiotic treatment. However, more data from low- and middle-income countries are needed to test this hypothesis.

## Data Availability

The original contributions presented in the study are included in the article/Supplementary Material, further inquiries can be directed to the corresponding authors.
